# Genetic overlap between schizophrenia and cognitive performance

**DOI:** 10.1038/s41537-024-00453-5

**Published:** 2024-03-05

**Authors:** Jianfei Zhang, Hao Qiu, Qiyu Zhao, Chongjian Liao, Yuxuan Guoli, Qi Luo, Guoshu Zhao, Nannan Zhang, Shaoying Wang, Zhihui Zhang, Minghuan Lei, Feng Liu, Yanmin Peng

**Affiliations:** 1 College of Computer and Control Engineering, Qiqihar University, Qiqihar, Heilongjiang China; 2 Department of Radiology and Tianjin Key Laboratory of Functional Imaging, Tianjin Medical University General Hospital, Tianjin, China; 3 School of Medical Imaging and Tianjin Key Laboratory of Functional Imaging, Tianjin Medical University, Tianjin, China; 4 The Second Hospital of Tianjin Medial University, Tianjin, China

**Keywords:** Diseases of the nervous system, Diseases

## Abstract

Schizophrenia (SCZ), a highly heritable mental disorder, is characterized by cognitive impairment, yet the extent of the shared genetic basis between schizophrenia and cognitive performance (CP) remains poorly understood. Therefore, we aimed to explore the polygenic overlap between SCZ and CP. Specifically, the bivariate causal mixture model (MiXeR) was employed to estimate the extent of genetic overlap between SCZ (*n* = 130,644) and CP (*n* = 257,841), and conjunctional false discovery rate (conjFDR) approach was used to identify shared genetic loci. Subsequently, functional annotation and enrichment analysis were carried out on the identified genomic loci. The MiXeR analyses revealed that 9.6 K genetic variants are associated with SCZ and 10.9 K genetic variants for CP, of which 9.5 K variants are shared between these two traits (Dice coefficient = 92.8%). By employing conjFDR, 236 loci were identified jointly associated with SCZ and CP, of which 139 were novel for the two traits. Within these shared loci, 60 exhibited consistent effect directions, while 176 had opposite effect directions. Functional annotation analysis indicated that the shared genetic loci were mainly located in intronic and intergenic regions, and were found to be involved in relevant biological processes such as nervous system development, multicellular organism development, and generation of neurons. Together, our findings provide insights into the shared genetic architecture between SCZ and CP, suggesting common pathways and mechanisms contributing to both traits.

## Introduction

Schizophrenia (SCZ) is a chronic psychiatric disorder, with an estimated lifetime prevalence of ~1%^1–3^. This disorder exerts a substantial impact on the individuals it affects, their families, and the larger societal context^4^. The associated symptoms, including hallucinations and delusions, can substantially disrupt daily routines, impeding one’s capacity to engage in work, education, and maintain interpersonal relationships, thereby resulting in noteworthy social and economic consequences^5^. Additionally, SCZ demonstrates a high heritability rate in twin studies, estimated at approximately 80%^6,7^, emphasizing the significant role of genetic factors in this disorder.

Cognitive dysfunction stands as a central and persistent facet of SCZ^8–14^, often encompassing memory deficits, thought disorganization, impaired attention, and difficulties in problem-solving^15,16^. There is evidence suggesting that cognitive impairments might emerge even before an official diagnosis of the condition^17–20^. Cognitive impairment is a prevalent feature among the majority of SCZ individuals, although its severity can differ from one person to another. In contrast to other aspects of SCZ, cognitive deficits often exhibit relative stability within the same patient over time, with their severity typically mirroring changes in the patient’s clinical condition^21^. Nevertheless, the fundamental pathophysiology of cognitive impairments in schizophrenia remains largely unknown, highlighting the crucial need to develop a deeper understanding of the underlying mechanisms causing these cognitive deficits.

The Psychiatric Genomics Consortium (PGC, http://www.med.unc.edu/pgc) conducted a genome-wide association study (GWAS) involving 76,755 SCZ patients and 243,649 controls, and found 287 risk loci associated with SCZ^22^. In parallel, GWAS was performed on a sample of over 250,000 individuals and identified 225 significant genome-wide loci associated with cognitive performance (CP)^23^. Accumulating evidence suggests genetic overlap between SCZ and cognitive functioning^24–26^. Moreover, a previous study utilizing the conditional/conjunctional false discovery rate (cond/conj FDR) method to reveal 21 shared loci between SCZ and cognitive domains^17^. These findings, though remarkable, originated from GWAS studies with relatively smaller sample sizes and limited discovery of significant shared loci, emphasizing the need for additional investigation and validation. Furthermore, MiXeR^27^, a recently proposed method, assesses genetic overlap regardless of the direction of effects and offers a comprehensive estimation of shared polygenic architecture. It also has the capability to calculate polygenicity, discoverability, and heritability of complex phenotypes, thus enhancing our comprehension of cross-trait genetic architectures. However, there have been no studies to date that have employed the MiXeR approach to investigate the genetic correlation between SCZ and CP.

In the current study, the primary objective was to examine the genetic overlap of SCZ and CP. By utilizing the recently published GWAS summary data^22,23^, the MiXeR approach was initially employed to estimate the genetic overlap between SCZ and CP, extending beyond the measurement of their genetic correlation^27^. Subsequently, the cond/conjFDR method^28^ was applied to identify the specific shared genetic loci^29^.

## Methods

### GWAS Data

All GWAS datasets used in this study were approved by the ethics committees of original studies, and all individuals provided informed consent prior to their participation. The GWAS summary statistics for SCZ were derived from the PGC, including data from 53,386 cases and 77,258 controls of European ancestry^22^. The GWAS summary statistics for CP were obtained from the Social Science Genetic Association Consortium (SSGAC) and encompassed 257,841 samples^23^. For details see Supplementary Methods and Supplementary Table 1.

### Statistical analysis of genetic overlap between SCZ and CP

To estimate the genetic overlap between SCZ and CP, MiXeR (https://github.com/precimed/mixer) was used, which employs GWAS summary statistics to quantify polygenic overlap^27^. MiXeR is capable of estimating the polygenicity, heritability, and discoverability of a single phenotype through univariate analysis^30^, as well as quantifying the genetic overlap of cross-traits. In the cross-trait analysis, the additive genetic effect was modeled as a mixture of four bivariate Gaussian components relating to the two traits. The performance of the model was evaluated using the negative log-likelihood function, and the best model was determined based on the comparison against those with the smallest and largest polygenic overlaps (lower scores indicating better performance). For more details on MiXeR, please refer to the Supplemental Methods. The enrichment of cross-traits was visualized through conditional *Q*-*Q* plots, where enrichment is observed when the proportion of SNPs associated with the primary phenotype increases with the strength of the secondary phenotype association^29^.

Afterwards, the condFDR/conjFDR method^28^ was employed to identify the shared genetic loci. By utilizing condFDR, we aimed to increase the discovery rate of genetic variants associated with SCZ and CP, enhancing the identification of genetic loci for major phenotypes by utilizing the association of both major and minor phenotypes^29^. Subsequently, conjFDR analysis was performed to identify genomic loci jointly associated with both traits, with conjFDR value was defined as the maximum of the two mutual condFDR values. In this study, SNPs below the thresholds of condFDR < 0.01 and conjFDR < 0.05 were considered significant. To minimize the inflation effect resulting from linkage disequilibrium (LD) dependency among SNPs, we performed 500 iterations of random pruning in the condFDR/conjFDR analysis. During each iteration, a single random SNP representative was preserved for each LD block, and the results from all iterations were subsequently averaged.

Notably, before performing MiXeR and condFDR/conjFDR analyses, genomic regions exhibiting LD were excluded^31^. Variants located in these specific genomic areas, such as the extended major histocompatibility complex (MHC) region (hg19 location Chr6: 25119106-33854733), chromosome 8p23.1 (hg19 location Chr8:7200000-12500000), and the gene MAPT (hg19 location Chr17:40000000-47000000), were removed^32^.

### Genomic loci definition

Functional Mapping and Annotation (FUMA, https://fuma.ctglab.nl/) was employed to identify independent genomic loci^33^, which serves as an online tool for functionally mapping genetic variants. Initially, independent significant SNPs were identified as SNPs that were independent from each other with *r*^2^ < 0.6 and condFDR < 0.01 or conjFDR < 0.05. From this set, we further selected lead SNPs by identifying a subset that showed LD with each other at *r*^2^ < 0.1.

### Functional annotation

All candidate SNPs from the genomic loci showing condFDR or conjFDR value < 0.1 and *r*^2^ ≥ 0.6 with one of the independent significant SNPs were utilized for functional annotation^33^. SNPs were annotated through various methods, incorporating combined annotation-dependent depletion (CADD) scores for predicting the deleterious effects of SNPs on protein structure and function^34^, RegulomeDB scores to assess the likelihood of regulatory functionality^35^, and chromatin states to predict transcriptional and regulatory effects within the vicinity of the SNP locus^36,37^. Finally, we utilized g: Profiler (https://biit.cs.ut.ee/gprofiler/gost) to evaluate gene-set enrichment for the genes located nearest to the identified shared loci, enhancing our understanding of the genetic mechanisms and potential underlying biological processes in these diseases^38^.

### Validation analyses

To evaluate the robustness and consistency of our MiXeR and cond/conjFDR findings, validation analyses were conducted using an additional CP dataset from Savage JE et al.^39^. Comprising 269,867 individuals from 14 cohorts, this dataset has been widely utilized in analogous multivariate genomic analyses^40,41^, offering an opportunity to validate our results derived from the SSGAC study^23^. Prior to initiating the validation analyses, LD was calculated between the lead SNPs of genome-significant loci reported in these original studies^23,39^. Subsequently, MiXeR and condFDR analyses were performed on the Savage et al. dataset using the same procedures as mentioned above. Finally, the estimates of genetic overlap between SCZ and CP obtained from MiXeR analysis were compared, and LD between the lead SNPs in the loci identified through cond/conjFDR analysis in both datasets was also calculated. In the validation analysis, LD with an *r*^2^ > 0.9 was considered indicative of replicability.

## Results

### Genetic overlap between SCZ and CP

**Fig. 1:**
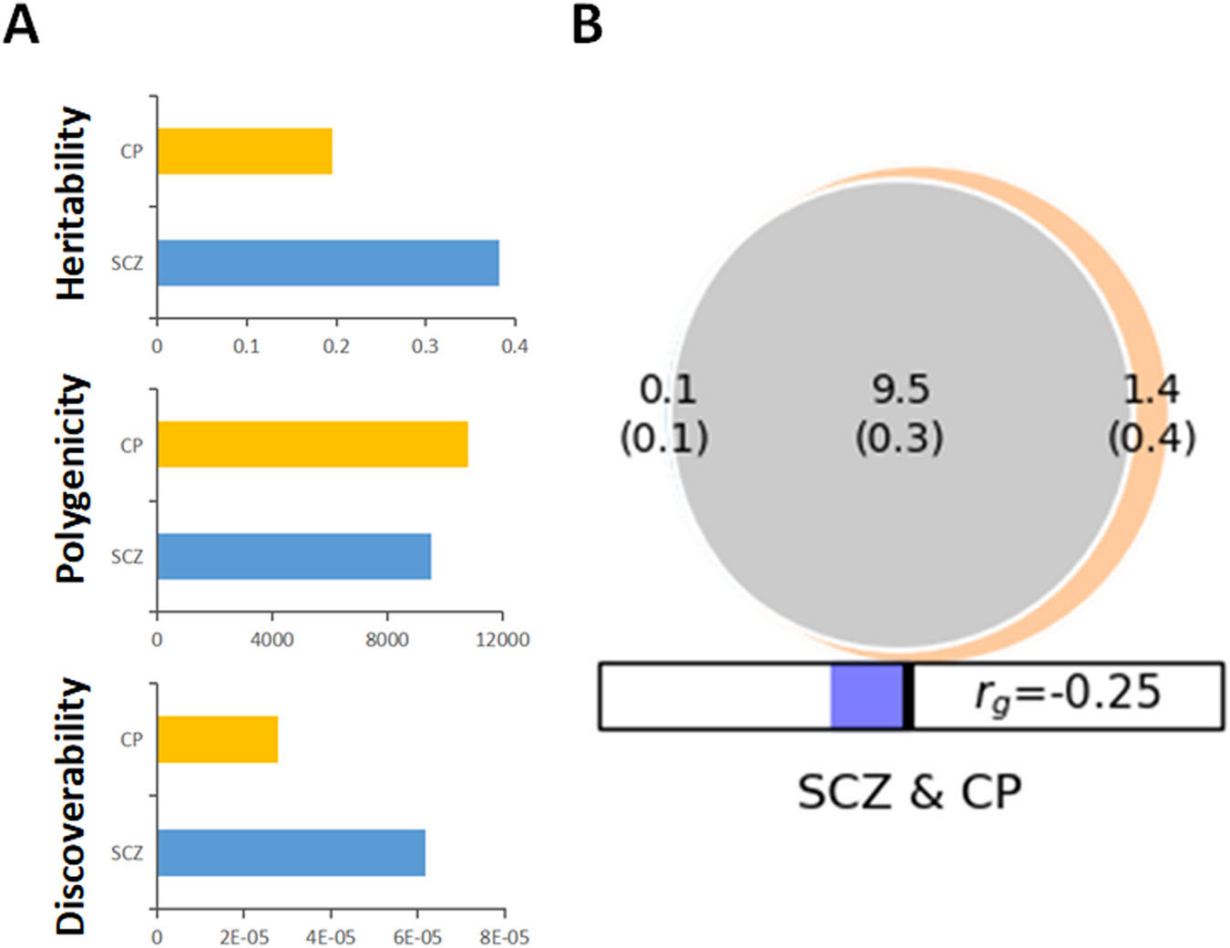
Shared and unique polygenic variants of SCZ and CP. **A** The MiXeR-estimated heritability, polygenicity, and discoverability for SCZ and CP. **B** Polygenic overlap between SCZ (blue) and CP (orange). The numbers in the Venn diagram indicate the estimated number of shared and specific genetic variants (in thousands), the numbers in parentheses are standard errors, and *r*_*g*_ corresponds to genetic correlation between two traits. SCZ schizophrenia, CP cognitive performance.

Univariate MiXeR analysis identified lower polygenicity (SCZ = 9572 SNPs, standard error = 255; CP = 10,874 SNPs, standard error = 297), higher heritability (SCZ = 0.39, standard error = 0.005; CP = 0.20, standard error = 0.002), and higher discoverability (SCZ = 6.28e-05, standard error = 1.47e-06; CP = 2.77e-05, standard error = 6.51e-07) for SCZ than CP (Fig. 1A). In bivariate MiXeR analysis, a genetic correlation of −0.25 was calculated between SCZ and CP (Fig. 1B), and a substantial genetic overlap was found, with a Dice coefficient of 92.8%. Specifically, there are about 9.5 K SNPs shared between SCZ and CP, representing 98.9% of the SNPs influencing SCZ and 87.1% of the SNPs influencing CP. Furthermore, there are 0.1 K SNPs (1.1%) uniquely associated with SCZ and 1.4 K SNPs (12.9%) only associated with CP. The substantial difference between the number of shared SNPs and those specific to each trait highlights a significant genetic overlap between SCZ and CP.

**Fig. 2:**
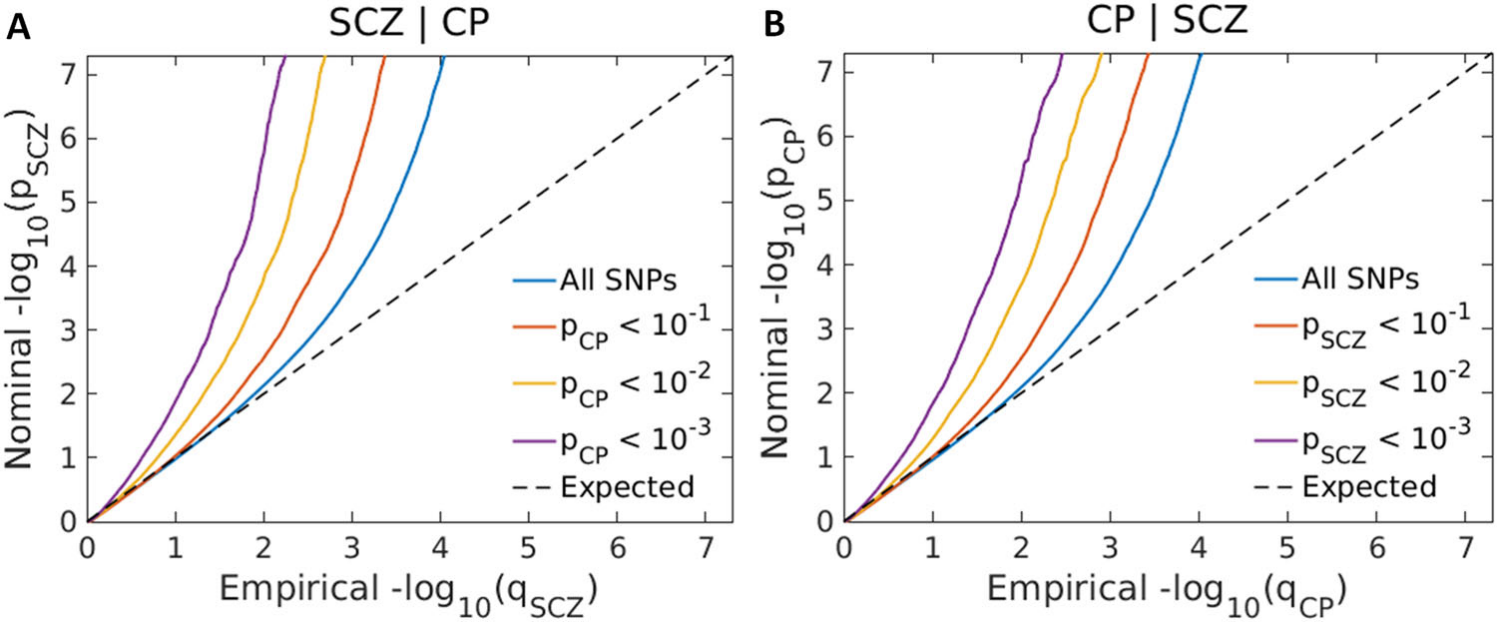
Polygenic overlapping effects of SCZ and CP. **A** Conditional *Q-Q* plots of nominal versus empirical -log10 *p*-values in SCZ conditional on CP and (**B**) vice versa, at the level of *p* < 0.100, *p* < 0.010 and *p* < 0.001, respectively. The blue line includes all SNPs and dashed lines indicate the null hypothesis. SCZ schizophrenia, CP cognitive performance.

The conditional *Q-Q* plots revealed an enrichment of SCZ and CP, indicating a polygenic overlap between them (Fig. 2A, B). In these plots, the blue line represents the association *p*-values of all SNPs in the primary trait, without considering their association with the conditional trait. The red line represents the association *p*-values of SNPs in the main trait that meet the condition of having the conditional trait association *p*-value less than 0.1. The yellow and purple lines represent the conditional trait association *p*-values of 0.01 and 0.001, respectively. As the threshold for the *p*-values of the conditional trait becomes stricter, the curve in the *Q-Q* plot continuously shifts towards the left, indicating the pleiotropic enrichment of SNP associations between SCZ and CP.

### Shared loci between SCZ and CP

**Fig. 3:**
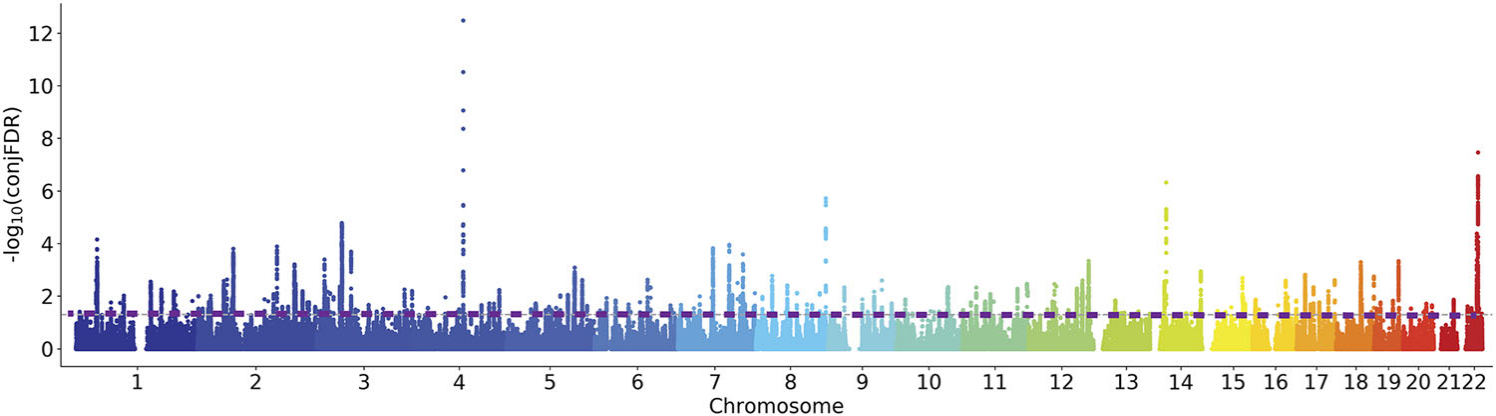
Shared genetic variants of SCZ and CP. The Manhattan plot shows the -log10 transformed conjFDR values for each SNP along the *Y*-axis and chromosomal positions along the *X*-axis. The lilac horizontal line represents the threshold for significant shared associations (conjFDR < 0.05, i.e., -log10(conjFDR) > 1.3).

**Fig. 4:**
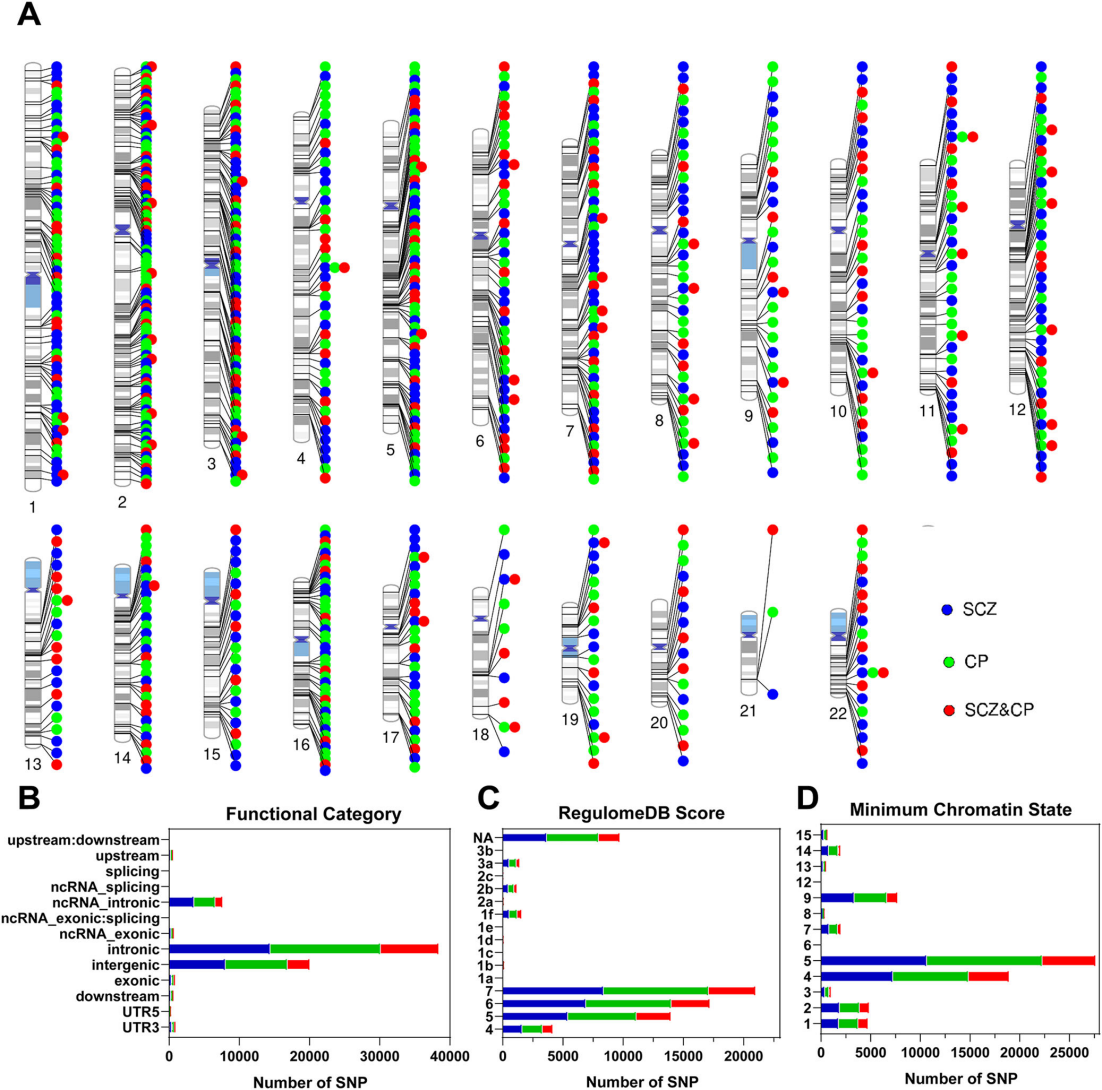
Distribution of loci and functional annotations of all candidate SNPs for SCZ and CP by condFDR/conjFDR analysis. **A** Distribution of 313 loci for SCZ, 332 loci for CP, and 236 loci shared between the two, spans across the genomic landscape of chromosomes. Distribution of (**B**) functional consequences, (**C**) RegulomeDB scores, and (**D**) minimum chromatin state of all candidate SNPs in loci for SCZ, loci for CP and loci shared between the two. The blue color represents SCZ trait conditioned on CP at condFDR < 0.01. The green color represents CP trait conditioned on SCZ at condFDR < 0.01. The red color represents shared associations between SCZ and CP at conjFDR < 0.05. SCZ schizophrenia, CP cognitive performance.

The condFDR analysis was employed to enhance the detection of genetic variants associated with SCZ and CP. At a condFDR < 0.01 threshold, we identified 313 loci associated with SCZ conditional on CP (Supplementary Table 2). Similarly, we found 332 loci associated with CP conditional on SCZ (Supplementary Table 3). Additionally, at a conjFDR < 0.05 threshold, we observed 236 gene loci that exhibited joint association with both SCZ and CP (Supplementary Table 4 and Fig. 3). Among these shared loci, 170 were not identified in the original GWAS for SCZ^22^, and 177 were not found in the original GWAS for CP^23^, with 139 newly identified shared loci for both SCZ and CP (Supplementary Table 4). The genomic distributions resulting from the condFDR/conjFDR analyses for SCZ and CP are shown in Fig. 4A. In the comparison of effect directions for lead SNPs at shared loci, we found that 60 (25.4%) lead SNPs exhibit consistent effect directions in SCZ and CP, while 176 (74.6%) lead SNPs show opposite effect directions (Supplementary Table 4).

### Functional annotation

Functional annotation of all candidate SNPs identified in the condFDR/conjFDR analysis was presented in Fig. 4B–D. The annotation of the candidate SNPs (*n* = 27,811) within the loci associated with SCZ conditional on CP revealed that the majority were situated in intronic regions (51.9%) and intergenic regions (29%), with only 1.3% located in exonic regions (Supplementary Table 5). Similarly, the annotation of the candidate SNPs (*n* = 29,413) within the loci associated with CP conditional on SCZ demonstrated that the majority were located in intronic regions (53.5%) and intergenic regions (30%), with only 1.2% found in exonic regions (Supplementary Table 6). When considering all candidate SNPs (*n* = 13,736) within the shared loci between SCZ and CP, the functional annotation indicated that the majority of these loci were situated in intergenic regions (23.3%) and intronic regions (60.5%), with 1.6% located in exonic regions (Supplementary Table 7).

Among the 236 top lead SNPs in the shared loci, 47.9% were located in intronic, 31.8% in intergenic, and 2.1% in exonic regions (Supplementary Table 4). Moreover, 17 of the 236 top lead SNPs had CADD scores above 12.37, suggesting a high level of deleteriousness. Five of these 17 SNPs (rs1506536, rs9878063, rs7708343, rs3789023, and rs2916142) were located in intronic regions within *MRPL33: BRE*, *CELSR3*, *FBXL17*, *MAPK8IP1*, and *PITPNC1*, respectively. Three of the 236 top lead SNPs (rs10774563, rs4275659, and rs5751191) had RegulomeDB scores of 1f, 1d, and 1f, respectively, indicating their potential influence on transcription factor binding; these SNPs annotated to RP11-728G15.1, ABCB9 and SEPT3 as nearest genes, respectively. The distribution of minimum chromatin state showed that 208 of the top lead SNPs were located in open chromatin states regions (states 1-7), indicating accessibility and openness of specific genome regions in cells. Furthermore, the gene-set enrichment analysis revealed that the shared loci between SCZ and CP were involved in biological processes such as nervous system development, multicellular organism development, and generation of neurons (Supplementary Table 8).

### Validation analyses

Validation analyses were conducted to assess the robustness and reliability of our findings. This involved comparing the MiXeR and cond/conjFDR results obtained from the SSGAC dataset^23^ used in our study with those from the dataset by Savage JE et al.^39^. The original GWAS findings from the SSGAC dataset indicated the presence of 225 distinct loci, while Savage JE et al. identified 205 loci in their corresponding study^23,39^. Subsequent LD analysis demonstrated that 80 loci were replicable in these two GWAS results, with LD *r*^2^ > 0.9.

In the Savage JE et al. dataset, the univariate MiXeR analysis showed that CP had 10,366 SNPs for polygenicity (standard error = 335), with a heritability of 0.18 (standard error = 0.002) and a discoverability of 2.64e-05 (standard error = 6.82e-07). The bivariate MiXeR analysis uncovered a genetic correlation of −0.24 and a Dice coefficient of 94.8%, with a polygenic overlap of 9.5 K observed between SCZ and CP (Supplementary Fig. 3). The consistency in polygenic overlap estimates (all 9.5 K SNPs for SSGAC and Savage JE et al.) and genetic correlation values (−0.25 for SSGAC, −0.24 for Savage JE et al.) highlights the robustness and reliability of our findings across different datasets, providing strong evidence for a shared genetic architecture between SCZ and CP.

In the validation analysis with condFDR < 0.01, 312 loci were identified for SCZ (Supplementary Table 9), and 314 loci were identified for CP (Supplementary Table 10). Among them, a total of 257 loci were replicable for SCZ (with 220 lead SNPs identical to those previously detected), and 146 loci were replicable for CP (with 79 lead SNPs identical to those previously detected) based on LD analysis (*r*^2^ > 0.9). Under conjFDR < 0.05, 238 shared loci were identified between SCZ and CP (Supplementary Table 11), and 128 loci were replicable (with 86 lead SNPs identical to those previously detected), with LD *r*^2^ > 0.9. These results demonstrate the moderate reproducibility of our findings.

## Discussion

In this study, we employed advanced analytical techniques to analyze large-scale GWAS datasets for SCZ and CP, aiming to comprehensively explore the shared genetic foundation between these traits. Specifically, utilizing MiXeR, we identified a substantial genetic overlap between these two traits. In addition, we uncovered 236 shared loci between SCZ and CP using conjFDR approach, including 139 newly identified loci. Functional annotation revealed their involvement in crucial biological processes, such as nervous system development and neuron generation. These findings shed light on the complex interplay between genetic factors underpinning SCZ and CP, advancing our understanding of the genetic mechanisms that shape these conditions.

The MiXeR method was utilized to identify mixed genetic effects and estimate the total number of causal variants. The results indicated a significant genetic overlap between SCZ and CP, with 9.5 K shared SNPs representing 98.9% of SCZ-influencing SNPs and 87.1% of CP-influencing SNPs. We also utilized the conjFDR approach to investigate the shared loci between SCZ and CP, revealing 236 shared loci. The concurrent use of these approaches allowed for a more precise and comprehensive assessment of genetic overlap among traits. However, the shared loci exhibit mixed effects, where opposing influences counteract each other (Supplementary Table 4), resulting in a modest genetic correlation between the two phenotypes. For the 74.6% of these 236 shared loci, we observed opposite genetic effects between SCZ and CP at these loci, suggesting that these variants may be contributing factors to the cognitive decline observed in individuals with SCZ. This mixed effect direction observed in the shared genetic loci of SCZ with CP further emphasizes the complexity of the genetic factors in influence.

Functional annotation demonstrates that the shared genetic variants between SCZ and CP are predominantly located in intronic and intergenic regions. Gene-set enrichment analysis implicates biological processes crucial for brain development, offering potential targets for therapeutic interventions aimed at preserving or enhancing cognitive function in individuals with schizophrenia. Interestingly, some of the genes identified in this study, such as *SP4*, *STAG1*, and *FAM120A*, had been previously reported to be associated with SCZ in other studies^42,43^. For example, the protein encoded by the *SP4* gene, a transcription factor capable of binding to the GC promoter regions of various genes, including those related to the photoreceptor signal transduction system, is linked to both schizophrenia^44^ and bipolar affective disorder^45^. Similarly, a search in the GWAS catalog confirmed that the genetic risk loci reported in this study had also been validated in previous research. For instance, rs4275659, identified as a shared SNP for SCZ and CP in this study, was found to jointly impact both phenotypes, consistent with reports on its effects on CP, education, and SCZ^46^. These findings emphasize the significance of the identified genetic factors and their potential role in contributing to the complex etiology of SCZ and related traits.

Several limitations should be taken into account when interpreting our findings. First, the availability of GWAS summary statistic data was confined to populations of European ancestry, potentially constraining the applicability of our findings to other populations. Subsequent investigations involving diverse ethnic backgrounds could provide insights into the broader genetic landscape of SCZ and CP. Second, our study primarily focused on genetic overlap, and the clinical implications or causal relationships between the identified genetic factors and observed phenotypes were not extensively explored. Future studies should consider incorporating additional clinical and environmental factors for a more comprehensive understanding. Finally, it is crucial to acknowledge that the observed moderate reproducibility in our validation analyses is a limitation rooted in the moderate replicability of the original CP GWAS datasets. To overcome this limitation, future research should prioritize the inclusion of larger and more diverse cognitive GWAS datasets.

In conclusion, this study highlights a significant genetic overlap between SCZ and CP, with ~9.5 K genetic variants influencing both SCZ and CP. Moreover, 236 shared genetic loci between these two traits were identified. These loci, predominantly located in intergenic and intronic regions, are associated with critical biological processes such as nervous system development and neuron generation. Overall, this study enriches our understanding of the complex relationship between SCZ and CP, offering valuable insights into potential molecular mechanisms, and providing a solid foundation for advancing research on schizophrenia treatment, prevention, and novel therapeutic interventions.

### Supplementary information

Supplementary Material

Supplementary Table 1-11

## Data Availability

Genome-wide association study summary statistics used in this study are publicly available from the Psychiatric Genetics Consortium (https://pgc.unc.edu/) and the Social Science Genetic Association Consortium (https://www.thessgac.org/).

## Code availability

This study employed already published approaches, the codes for which are accessible on public repositories: MiXeR (https://github.com/precimed/mixer), cFDR (https://github.com/precimed/pleiofdr/), FUMA (http://fuma.ctglab.nl/) and g:Profiler (https://biit.cs.ut.ee/gprofiler/gost).

## References

[1] Ahangari M (2022). Genome-wide analysis of schizophrenia and multiple sclerosis identifies shared genomic loci with mixed direction of effects. Brain Behav. Immun..

[2] Saha S, Chant D, McGrath J (2007). A systematic review of mortality in schizophrenia: is the differential mortality gap worsening over time?. Arch. Gen. Psychiatry.

[3] Lam M (2019). Comparative genetic architectures of schizophrenia in East Asian and European populations. Nat. Genet..

[4] Jongsma HE, Turner C, Kirkbride JB, Jones PB (2019). International incidence of psychotic disorders, 2002-17: a systematic review and meta-analysis. Lancet Public Health.

[5] Jin H, Mosweu I (2017). The societal cost of schizophrenia: a systematic review. Pharmacoeconomics.

[6] Focking M (2019). Epigenetic factors in schizophrenia: mechanisms and experimental approaches. Mol. Neuropsychiatry.

[7] Cheng W (2021). Genetic association between schizophrenia and cortical brain surface area and thickness. JAMA Psychiatry.

[8] Smeland OB, Andreassen OA (2018). How can genetics help understand the relationship between cognitive dysfunction and schizophrenia?. Scand. J. Psychol..

[9] Green MF, Harvey PD (2014). Cognition in schizophrenia: past, present, and future. Schizophr Res. Cogn..

[10] Simonsen C (2011). Neurocognitive dysfunction in bipolar and schizophrenia spectrum disorders depends on history of psychosis rather than diagnostic group. Schizophr. Bull..

[11] Green MF, Kern RS, Braff DL, Mintz J (2000). Neurocognitive deficits and functional outcome in schizophrenia: are we measuring the “right stuff”?. Schizophr. Bull..

[12] Abplanalp SJ (2023). A Bayesian network approach to social and nonsocial cognition in schizophrenia: are some domains more fundamental than others?. Schizophr Bull.

[13] Green MF, Horan WP, Lee J (2019). Nonsocial and social cognition in schizophrenia: current evidence and future directions. World Psychiatry.

[14] Haddad C, Salameh P, Sacre H, Clement JP, Calvet B (2023). Effects of antipsychotic and anticholinergic medications on cognition in chronic patients with schizophrenia. BMC Psychiatry.

[15] Dickinson D, Ragland JD, Gold JM, Gur RCJBp. (2008). General and specific cognitive deficits in schizophrenia: Goliath defeats David?. Biol. Psychiatry.

[16] Aas M, Dazzan P, Mondelli V, Melle I, Murray RM (2014). A systematic review of cognitive function in first-episode psychosis, including a discussion on childhood trauma, stress, and inflammation. Front. Psychiatry.

[17] Smeland OB (2017). Identification of genetic loci jointly influencing schizophrenia risk and the cognitive traits of verbal-numerical reasoning, reaction time, and general cognitive function. JAMA Psychiatry.

[18] Mallet J, Le Strat Y, Dubertret C, Gorwood P (2020). Polygenic risk scores shed light on the relationship between schizophrenia and cognitive functioning: review and meta-analysis. J. Clin. Med..

[19] Mesholam-Gately RI, Giuliano AJ, Goff KP, Faraone SV, Seidman LJ (2009). Neurocognition in first-episode schizophrenia: a meta-analytic review. Neuropsychology.

[20] Ohi K (2018). Genetic overlap between general cognitive function and schizophrenia: a review of cognitive GWASs. Int. J. Mol. Sci..

[21] Bowie CR, Harvey PD (2006). Cognitive deficits and functional outcome in schizophrenia. Neuropsychiatr. Dis. Treat..

[22] Trubetskoy V (2022). Mapping genomic loci implicates genes and synaptic biology in schizophrenia. Nature.

[23] Lee JJ (2018). Gene discovery and polygenic prediction from a genome-wide association study of educational attainment in 1.1 million individuals. Nat. Genet..

[24] Hubbard L (2016). Evidence of common genetic overlap between schizophrenia and cognition. Schizophr. Bull..

[25] Lencz T (2014). Molecular genetic evidence for overlap between general cognitive ability and risk for schizophrenia: a report from the Cognitive Genomics consorTium (COGENT). Mol. Psychiatry.

[26] Toulopoulou T (2007). Substantial genetic overlap between neurocognition and schizophrenia: genetic modeling in twin samples. Arch. Gen. Psychiatry.

[27] Frei O (2019). Bivariate causal mixture model quantifies polygenic overlap between complex traits beyond genetic correlation. Nat. Commun..

[28] Smeland OB (2020). Discovery of shared genomic loci using the conditional false discovery rate approach. Hum. Genet..

[29] Andreassen OA (2013). Improved detection of common variants associated with schizophrenia and bipolar disorder using pleiotropy-informed conditional false discovery rate. PLoS Genet..

[30] Holland D (2020). Beyond SNP heritability: polygenicity and discoverability of phenotypes estimated with a univariate Gaussian mixture model. PLoS Genet..

[31] Schwartzman A, Lin X (2011). The effect of correlation in false discovery rate estimation. Biometrika.

[32] Karadag N (2023). Identification of novel genomic risk loci shared between common epilepsies and psychiatric disorders. Brain.

[33] Watanabe K, Taskesen E, van Bochoven A, Posthuma D (2017). Functional mapping and annotation of genetic associations with FUMA. Nat. Commun..

[34] Kircher M (2014). A general framework for estimating the relative pathogenicity of human genetic variants. Nat. Genet..

[35] Boyle AP (2012). Annotation of functional variation in personal genomes using RegulomeDB. Genome Res. Sep..

[36] Roadmap Epigenomics Consortium (2015). Integrative analysis of 111 reference human epigenomes. Nature.

[37] Zhu Z (2016). Integration of summary data from GWAS and eQTL studies predicts complex trait gene targets. Nat. Genet..

[38] Kolberg L (2023). g:Profiler-interoperable web service for functional enrichment analysis and gene identifier mapping (2023 update). Nucleic Acids Res..

[39] Savage JE (2018). Genome-wide association meta-analysis in 269,867 individuals identifies new genetic and functional links to intelligence. Nat. Genet..

[40] Hindley G (2023). Multivariate genetic analysis of personality and cognitive traits reveals abundant pleiotropy. Nat. Hum. Behav..

[41] Holen B (2023). Genome-wide analyses reveal shared genetic architecture and novel risk loci between opioid use disorder and general cognitive ability. Drug Alcohol Depend..

[42] Singh T (2022). Rare coding variants in ten genes confer substantial risk for schizophrenia. Nature.

[43] Schizophrenia Working Group of the Psychiatric Genomics Consortium. (2014). Biological insights from 108 schizophrenia-associated genetic loci. Nature.

[44] Zhou X (2022). Over-representation of potential SP4 target genes within schizophrenia-risk genes. Mol Psychiatry.

[45] Zhou X (2009). Transcription factor SP4 is a susceptibility gene for bipolar disorder. PLoS ONE.

[46] Lam M (2019). Pleiotropic meta-analysis of cognition, education, and schizophrenia differentiates roles of early neurodevelopmental and adult synaptic pathways. Am. J. Hum. Genet..

