# Supplementary material for: Genetic overlap between schizophrenia and cognitive performance
Source: Schizophrenia (Heidelb). 2024 Mar 5;10(1):31. doi: 10.1038/s41537-024-00453-5 (PMC10914834; doi:10.1038/s41537-024-00453-5)
Supplement: Supplementary file 1 — Supplementary Material [file 41537_2024_453_MOESM1_ESM.docx]

**Supplementary information**

**Supplementary Methods**

**Participants**

We utilized summary statistics from GWAS for our analysis. The data on schizophrenia (SCZ) were sourced from the Psychiatric Genomics Consortium (PGC) and included a total of 76,755 cases and 243,649 controls^1^. Specifically, we focused on samples of European ancestry within these cohorts, amounting to 53,386 cases and 77,258 controls. Furthermore, cognitive performance (CP) data were obtained from the Social Science Genetic Association Consortium (SSGAC), including the COGENT cohort (*n* = 35,298) and the UK Biobank cohort (*n* = 222,543), totaling 257,841 samples^2^ (Supplementary Table 1). It is important to note that there was no overlap in samples between the SCZ and CP datasets.

In the COGENT study, the cognitive phenotype for all 35 participating cohorts was defined as the first principal component derived from three or more neuropsychological tests, with specific tests varying among cohorts as outlined in the original study. In the UK Biobank analyses, the approach from prior research was adopted, considering the respondent’s score on a verbal-numerical reasoning test as the cognitive phenotype. The test consisted of thirteen logic and reasoning questions, each with a two-minute time limit, designed to assess fluid intelligence. Respondents had the opportunity to take the test up to four times. In the GWAS analysis, the cognitive performance phenotype is determined by calculating the mean of standardized scores across all test instances for each respondent.

**MiXeR**

MiXeR was used to assess the polygenic overlap for cross traits by analyzing GWAS summary statistics^3, 4^. In univariate MiXeR, $\beta_{j}=\pi_{0}N(0,0)+\pi_{1}N(0,\sigma_{\beta}^{2})$, for each SNP there is a $\beta_{j}$, $\pi_{0}$ represents the proportion of SNPS not associated with the phenotype, $\pi_{1}$(polygenic) represents the proportion of SNPS associated with the phenotype, and $\sigma_{\beta}^{2}$ represents the variance of the effect size of SNPS associated with the phenotype (discoverability). In the analysis of cross traits, MiXeR models additive genetic effects as a mixture of four binary Gaussian components about the two traits, $(\beta_{1j},\beta_{2j})=\pi_{0}N(0,0)+\pi_{1}N(0,\Sigma_{1})+\pi_{2}N(0,\Sigma_{2})+\pi_{12}N(0,\Sigma_{12})$, $\pi_{1}$ represents the weight of variants affecting only the first trait, $\pi_{2}$ represents the weight of variants affecting only the second trait, $\pi_{12}$ represents the weight of variants affecting both traits, and $\pi_{0}$ represents the weight of variants affecting no traits. Σ_12_$=\left[ \begin{matrix} \sigma_{1}^{2} & \rho_{12}\sigma_{1}\sigma_{2} \\ \rho_{12}\sigma_{1}\sigma_{2} & \sigma_{2}^{2} \end{matrix} \right]$ is a variance-covariance matrix, $\rho_{12}$ represents the correlation of effect size in the shared component, $\sigma_{1}^{2},\sigma_{2}^{2}$ and represents the discoverability parameter estimated for the two traits in the univariate analysis.

A negative log-likelihood plot can be used to evaluate the model’s performance in three scenarios: the best model, the model with the least polygenic overlap, and the model with the most overlap among multiple genes. The best model reflects the optimal polygenic overlap according to MiXeR, the smallest model has minimal overlap, and the largest model exhibits maximal overlap. In the negative log-likelihood plot (Supplementary Figures 1), the leftmost point represents the scenario with the least overlap, the rightmost point signifies the most overlap, and the lowest point on the curve indicates the best-fitting model.

**Conditional *Q-Q* plot**

A *Q-Q* plot is a tool used to compare the expected (nominal) probability distribution with the observed empirical distribution. Conditional on the null hypothesis, a line is formed with the corresponding value of the empirical distribution as the *X*-axis and the nominal *p*-value as the *Y*-axis. A stratified *Q-Q* plot is constructed by constraining the *p*-value for the secondary phenotype to more stringent thresholds such as *p* < 0.1, *p* < 0.01, and *p* < 0.001. As these thresholds become stricter, the *Q-Q* plot’s curve consistently shifts to the left, indicating a form of “enrichment” under the null hypothesis.

**Conditional and conjunctional FDR**

The conditional FDR (condFDR) is an extension of the standard FDR and takes into account information from the GWAS summary statistics of a second trait to adjust its level of significance. CondFDR is defined as the probability that an SNP has no association with the primary trait when the *p*-values for both the primary and secondary traits are as small as or smaller than the observed values. When ranking SNPs using either the standard FDR or *p*-values, the order remains the same. However, when the primary and secondary traits are genetically linked, condFDR rearranges the SNP ranking, resulting in a different order compared to using *p*-values alone. The conjunctional FDR (conjFDR) is defined as the posterior probability that an SNP has no association with either trait or both simultaneously, given that its *p*-values for both traits are as small as or smaller than the observed values. A conservative estimate of conjFDR can be obtained by taking the maximum condFDR for a specific SNP after performing the condFDR procedure for both traits and interchanging their roles.


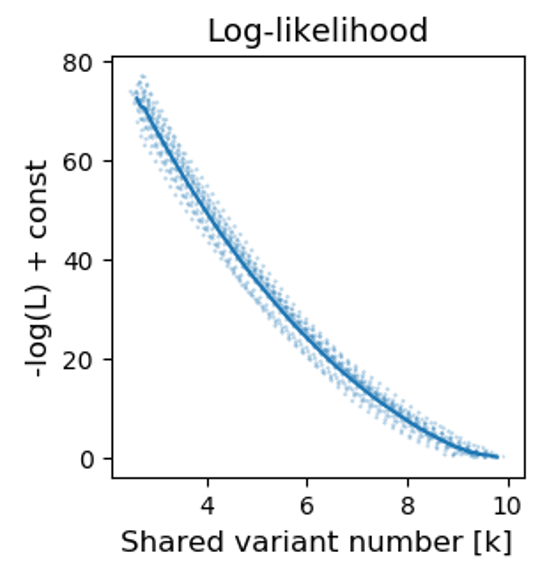


**Supplementary Figure 1.** Negative log-likelihood function plots of the genetic overlap of schizophrenia (SCZ) and cognitive performance (CP).


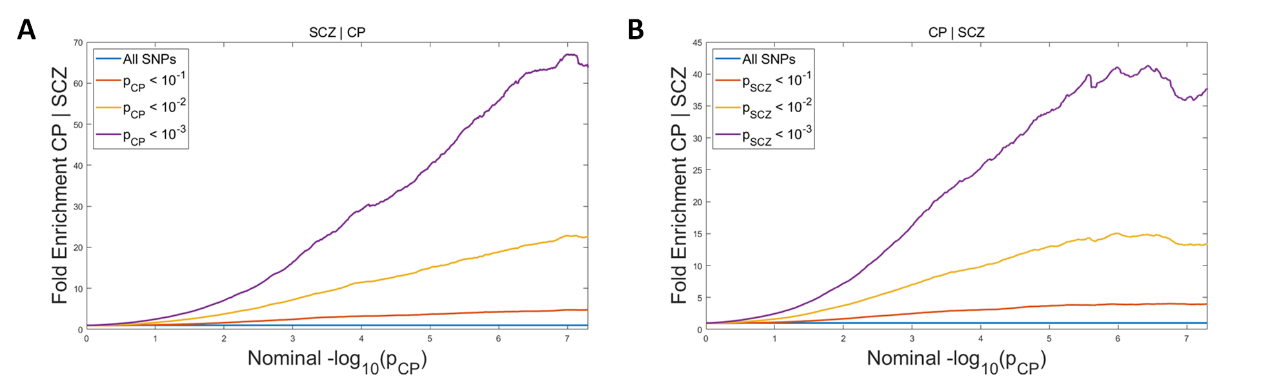


**Supplementary Figure 2.** Fold-enrichment plot for SCZ and CP. In the fold-enrichment plot, the *X*-axis represents nominal −log10 *p*-values, and the *Y*-axis represents the ratio of the cumulative distribution of −log10 *p*-values within a specific stratum to the cumulative distribution of all SNPs.

**
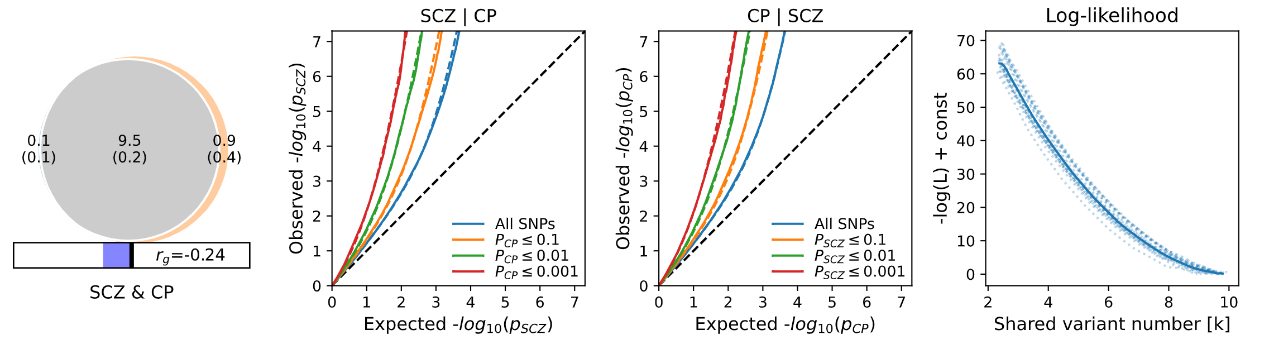
**

**Supplementary Figure 3.** Venn diagrams, conditional *Q-Q* plots, and negative log-likelihood function plots of the genetic overlap of schizophrenia (SCZ) and general cognitive ability (CP) in validation analysis. Venn diagrams shows the polygenic overlap (gray) between schizophrenia (blue) and general cognitive ability (orange), where the numbers in the figure represent the number (in thousands) of variants affecting the corresponding trait, and the numbers in parentheses represent standard errors. Conditional *Q-Q* plots, where *p*-values observed and expected for major traits (schizophrenia) are formed under the condition that secondary traits (general cognitive ability) are *p* < 0.1, *p* < 0.01, *p* < 0.001. The blue solid line represents all SNPs, the black dashed line represents the null hypothesis, and the blue, orange, green, and red dashed lines represent the model prediction curves. Negative log-likelihood plot, the smallest model is represented by the leftmost point in the figure, the largest model by the rightmost point in the figure, and the best model by the lowest point of the curve in the figure.

**References**

**1.** Trubetskoy V, Pardinas AF, Qi T, et al. Mapping genomic loci implicates genes and synaptic biology in schizophrenia. *Nature* Apr 2022;604(7906):502-508.

**2.** Lee JJ, Wedow R, Okbay A, et al. Gene discovery and polygenic prediction from a genome-wide association study of educational attainment in 1.1 million individuals. *Nat Genet* Jul 23 2018;50(8):1112-1121.

**3.** Holland D, Frei O, Desikan R, et al. Beyond SNP heritability: Polygenicity and discoverability of phenotypes estimated with a univariate Gaussian mixture model. *PLoS Genetics* 2020;16(5):e1008612.

**4.** Frei O, Holland D, Smeland OB, et al. Bivariate causal mixture model quantifies polygenic overlap between complex traits beyond genetic correlation. *Nature communications* 2019;10(1):2417.
